# Modulatory Effects of Monoamines and Perineuronal Nets on Output of Cerebellar Purkinje Cells

**DOI:** 10.3389/fncir.2021.661899

**Published:** 2021-06-14

**Authors:** Moritoshi Hirono, Fuyuki Karube, Yuchio Yanagawa

**Affiliations:** ^1^Department of Physiology, Wakayama Medical University, Wakayama, Japan; ^2^Lab of Histology and Cytology, Graduate School of Medicine, Hokkaido University, Sapporo, Japan; ^3^Department of Genetic and Behavioral Neuroscience, Gunma University Graduate School of Medicine, Maebashi, Japan

**Keywords:** Deep cerebellar nuclei, serotoinin, eyeblink conditioning, axon collateral, noradrenaline, Lugaro cell, globular cell, chondroitinase ABC

## Abstract

Classically, the cerebellum has been thought to play a significant role in motor coordination. However, a growing body of evidence for novel neural connections between the cerebellum and various brain regions indicates that the cerebellum also contributes to other brain functions implicated in reward, language, and social behavior. Cerebellar Purkinje cells (PCs) make inhibitory GABAergic synapses with their target neurons: other PCs and Lugaro/globular cells via PC axon collaterals, and neurons in the deep cerebellar nuclei (DCN) via PC primary axons. PC-Lugaro/globular cell connections form a cerebellar cortical microcircuit, which is driven by serotonin and noradrenaline. PCs’ primary outputs control not only firing but also synaptic plasticity of DCN neurons following the integration of excitatory and inhibitory inputs in the cerebellar cortex. Thus, strong PC-mediated inhibition is involved in cerebellar functions as a key regulator of cerebellar neural networks. In this review, we focus on physiological characteristics of GABAergic transmission from PCs. First, we introduce monoaminergic modulation of GABAergic transmission at synapses of PC-Lugaro/globular cell as well as PC-large glutamatergic DCN neuron, and a Lugaro/globular cell-incorporated microcircuit. Second, we review the physiological roles of perineuronal nets (PNNs), which are organized components of the extracellular matrix and enwrap the cell bodies and proximal processes, in GABA release from PCs to large glutamatergic DCN neurons and in cerebellar motor learning. Recent evidence suggests that alterations in PNN density in the DCN can regulate cerebellar functions.

## Introduction

**FIGURE 1:**
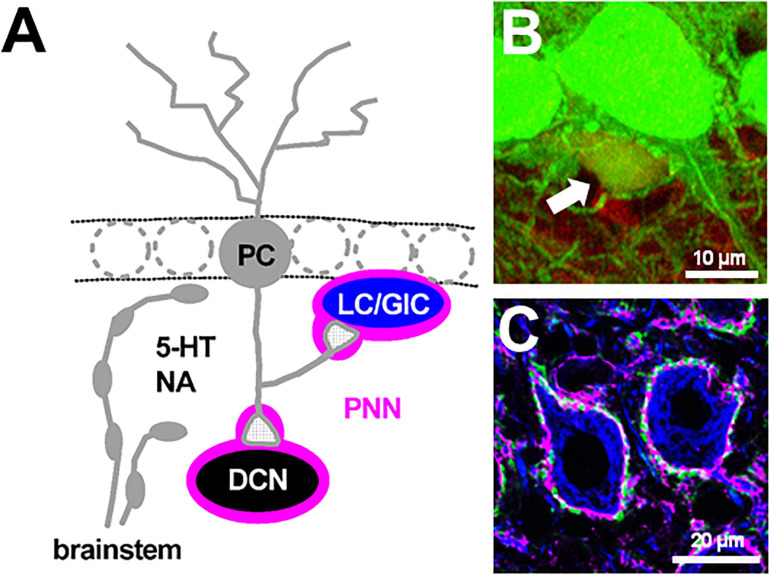
Perineuronal net-expressing cerebellar neurons targeted by Purkinje cells. **(A)** A Purkinje cell (PC) axon collateral makes GABAergic synapses with a Lugaro/globular cell (LC/GlC). A PC primary axon innervates a large glutamatergic neuron in the deep cerebellar nuclei (DCN). These PC target neurons are enwrapped by perineuronal nets (PNNs). 5-HT and NA are released from their beaded afferent fibers originated from the brainstem. **(B)** A GlC in the GAD67^+/GFP^ mouse cerebellum shows immunoreactivity for anti-calretinin antibody (red), thus its cell body is show in yellow (indicated by an arrow). **(C)** Cell bodies of large glutamatergic DCN neurons are immunolabeled by SMI32 antibodies (blue) and surrounded by PNNs as shown by *Wisteria floribunda agglutinin*-staining (magenta) and PC axon terminals, which show immunoreactivity against calbindin (green). **(B)** is from Hirono et al. (2012). **(C)** is our unpublished data.

Although the cerebellum is well known to play a crucial role in fine movement and motor activity (Ito, 1984), recent evidence suggest that the cerebellum is increasingly implicated in a variety of brain functions, including reward prediction (Wagner et al., 2017; Heffley et al., 2018; Carta et al., 2019; Kostadinov et al., 2019), motor planning (Gao et al., 2018; Chabrol et al., 2019; Wagner et al., 2019), and higher cognitive functions such as language and social behavior (Fiez and Petersen, 1998; Van Overwall et al., 2014; Schmahmann et al., 2019). Although the novel neural connections between the cerebellum and other brain areas and individual cellular elements are being unveiled rapidly (Judd et al., 2020; Kebschull et al., 2020; Kozareva et al., 2020), to achieve a comprehensive understanding of cerebellar functions, characterization of synaptic transmission and modulatory actions on neuronal and synaptic factors in the cerebellum is required. Inhibitory GABAergic transmission from Purkinje cells (PCs), the sole output neuron of the cerebellar cortex, has not been fully understood. This is because *in vitro* studies using cerebellar brain slices are mostly accompanied by PCs with damaged or cut axons, and it is difficult in identifying their target neurons among the numerous heterogeneous interneurons in the cerebellar cortex (Lainé and Axelrad, 2002; Crook et al., 2007; Simat et al., 2007; Schilling et al., 2008; Hirono, 2016; Prestori et al., 2019). In the historical view of the canonical neuronal network of the cerebellum, PCs project their primary axons to neurons in the deep cerebellar nuclei (DCN; Figure 1A): the fastigial (medial), interpositus, and dentate (lateral) (Ito et al., 1964; Obata et al., 1967; Chan-Palay, 1977; De Zeeuw and Berrebi, 1996; Mouginot and Gähwiler, 1995; Teune et al., 1998; Gauck and Jaeger, 2000; Najac and Raman, 2015). PC-mediated GABAergic inhibition regulates the rate and timing of DCN excitatory neuron output to cortical targets (Shin and De Schutter, 2006; Shin et al., 2007). DCN neurons produce the final output of the cerebellum by integrating the inhibitory inputs with excitatory inputs from mossy and climbing axon collaterals (Gauck and Jaeger, 2000; Rowland and Jaeger, 2005; Bengtsson et al., 2011; Steuber and Jaeger, 2013; Bengtsson and Jörntell, 2014). In the cerebellar cortex, the neuronal types targeted by PC axon collaterals have been controversial. PC axon collaterals have been suggested to form functional synaptic contacts with other PCs during early mouse development (Orduz and Llano, 2007; Watt et al., 2009). Recent electrophysiological studies demonstrated that even in adult mice, PC axon collaterals form synapses onto all PCs, Lugaro cells, globular cells (a subgroup of Lugaro cells) (Figures 1A,B), and one third of molecular layer interneurons (Hirono et al., 2012; Witter et al., 2016). Additionally, PCs form synaptic contacts with granule cells in a lobule-dependent manner (Guo C. et al., 2016). Therefore, it is important to characterize GABAergic transmission from PCs and their neuronal modulation.

The cerebellum receives beaded fibers, including various amines and peptides, such as serotonin (5-HT) and noradrenaline (NA) (Ito, 2009; Figure 1A). Serotonergic beaded fibers, the third largest population of afferent fibers into the cerebellum only to mossy and climbing fibers, arise from the raphe nuclei and the gigantocellular reticular formation adjacent to the raphe nuclei, forming dense plexuses in the granule cell layer and the PC layer (Bishop and Ho, 1985; Kerr and Bishop, 1991; Longley et al., 2020). A dense plexus of serotonergic fibers extending from various brainstem nuclei is also present in the DCN, however, they are not collaterals of the fibers innervating into the cortex (Kitzman and Bishop, 1994), suggesting that the cerebellar cortex and DCN could be controlled independently by serotonergic signals. By contrast, noradrenergic neurons in the brainstem nucleus, locus coeruleus, project their beaded afferent fibers not only to the cerebellar cortex, which are densely expressed in the granule cell layer especially under the PC layer (Nelson et al., 1997), but also to the DCN (Hökfelt and Fuxe, 1969; Olson and Fuxe, 1971; Siggins et al., 1971; Nedelescu et al., 2017). Although cerebellar motor learning is affected by 5-HT (Miyashita and Watanabe, 1984; Trouillas et al., 1995; Oades et al., 2008; Frings et al., 2010) and NA (Bickford et al., 1992; Paredes et al., 2009; Wakita et al., 2017), there has been few evidence for their modulatory action on GABAergic transmission from PCs and intrinsic neuronal excitability of Lugaro/globular cells and large glutamatergic DCN neurons. Therefore, first, we introduce the characteristics of GABAergic transmission at synapses between PC axon collaterals and Lugaro/globular cells, and PC primary axons and large glutamatergic neurons in the DCN, focusing on 5-HT- and NA-mediated modulatory effects on GABAergic transmission.

In the mature central nervous system (CNS), perineuronal nets (PNNs), which are extracellular matrices enwrapping the cell bodies and proximal processes of neurons, form ladder-like structures and restrict the structural neuronal plasticity (Fawcett et al., 2019; Reichelt et al., 2019). It has been reported that PNNs not only control excitability of neurons and synaptic activity as a neuromodulator (Dityatev and Rusakov, 2011; Balmer, 2016), but also regulate action of other neuromodulators by changing their diffusion in the extracellular space (Sylantyev et al., 2008; Gundelfinger et al., 2010). Interestingly, PC target neurons, Lugaro/globular cells in the cerebellar cortex and large DCN neurons, express the formation of PNNs around their soma and proximal processes, where PC terminals innervated predominantly (Figure 1; De Zeeuw and Berrebi, 1996; Chan-Palay, 1997; Uusissari and Knöpfel, 2008). Thus, second, we especially focus on the physiological significance of PNNs, which are expressed in the DCN and enwrap synapses between PCs and the large DCN neurons, in dynamic regulation of GABAergic transmission as well as in cerebellar motor learning.

## Lugaro/Globular Cells in the Cerebellar Cortex

### Morphological Characteristics and Incorporated Microcircuit of Lugaro/Globular Cells

Inhibitory interneurons in the cerebellar cortex are more heterogeneous than knowledge in traditional categorization (Lainé and Axelrad, 2002; Crook et al., 2006; Simat et al., 2007; Schilling et al., 2008; Hirono, 2016; Prestori et al., 2019; Kozareva et al., 2020). Lugaro cells, which were reported for the first time in the cat cerebellum, have unique morphological characteristics and are located just below PCs in the granular cell layer, or within the PC layer (Lugaro, 1894). Their typical cell bodies are spindle-shaped and dendrites project on both sides of the cell bodies oriented in the parasagittal plane (Lugaro, 1894; Fox, 1959). Their axons project into the molecular layer and then travel long in the mediolateral axis alongside parallel fibers (Palay and Chan-Palay, 1974; Lainé and Axelrad, 1996). Lugaro cells are glycinergic/GABAergic interneurons and their number is very low, i.e., it is one fifteenth of that of PCs in the rat cerebellar cortex (Sahin and Hockfield, 1990; Dieudonné and Dumoulin, 2000). Lugaro cells are mainly divided into two subgroups on the basis of their morphology: fusiform Lugaro cells within the granule cell layer (Lugaro, 1894; Geurts et al., 2001; Crook et al., 2006) and globular cells, whose cell body is small and globular-shaped and located under the PC layer (Lainé and Axelrad, 2002; Simat et al., 2007; Schilling et al., 2008; Hirono et al., 2012). Immunohistochemical staining with antibodies against Kv4.3, mGluR1α, Rat303, SMI311, and chondroitin sulfate proteoglycans (CSPGs) has been performed for detailed anatomical studies of Lugaro/globular cells (Sahin and Hockfield, 1990; Geurts et al., 2001; Hsu et al., 2003; Víg et al., 2003; Crook et al., 2007). A novel reporter mouse line, which expresses Yellow Cameleon preferentially in Lugaro/globular cells, demonstrated their dendritic and axonal arborizations in relation to cerebellar compartments (Miyazaki et al., 2020). Furthermore, a recent transcriptomic study suggested cell type markers for putative Lugaro cells (*Htr2a* or *Edil3*) and for putative globular cells (*Aldh1a3* or *Slc6a5*) (Kozareva et al., 2020).

In the parasagittal plane, axons of Lugaro/globular cells make synapses with basket/stellate cells (Lainé and Axelrad, 1998), and the transverse axons of Lugaro/globular cells innervate Golgi cells (Dieudonné and Dumoulin, 2000; Dumoulin et al., 2001; Hirono et al., 2012). Inhibitory synaptic signals from approximately 10 Lugaro/globular cells converge on one Golgi cell. One Lugaro/globular cell presumably forms divergent contacts with approximately 150 Golgi cells (Dieudonné and Dumoulin, 2000; Dieudonné, 2001; Dumoulin et al., 2001), suggesting that Lugaro/globular cells can synchronize activity among Golgi cells.

**FIGURE 2:**
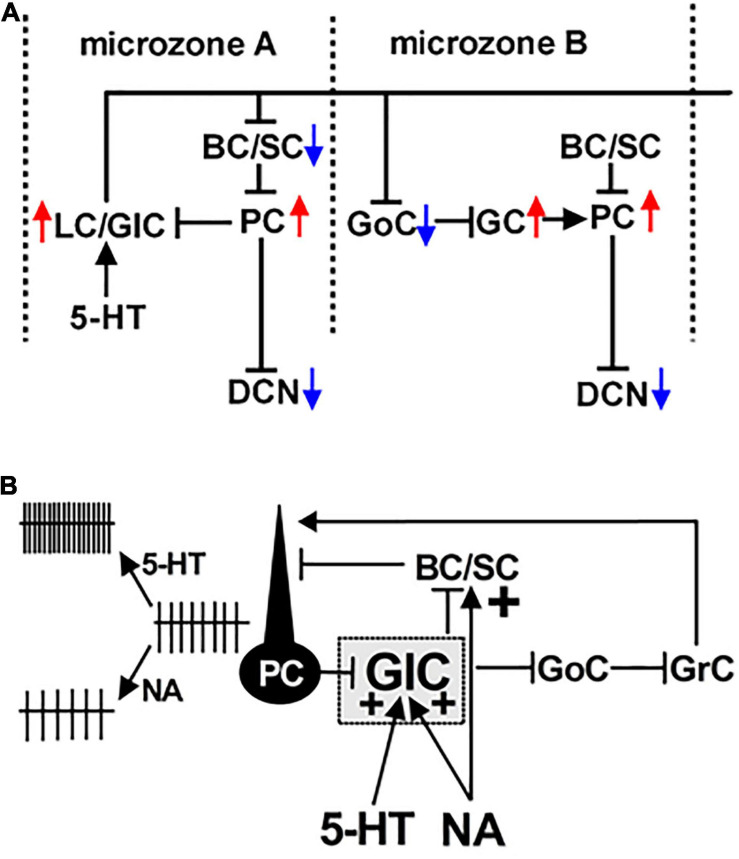
Schematic representation of a Lugaro/globular cell-incorporated microcircuit. **(A)** The functional connection between a Purkinje cell (PC) and a Lugaro/globular cell (LC/GlC) is represented in microzone A. The LC/GlC forms synaptic connections with a basket/stellate cell (BC/SC) in the sagittal plane and project its long and transversal axons, which intersect several microzones. Its axon collaterals form synaptic contacts with Golgi cells (GoCs) in microzone B. Thus, firing of the LC/GlC likely synchronize firing of PC groups between different microzones. Red and blue arrows denote excitation and hyperpolarization of neurons, respectively. **(B)** A microcircuit equipped with PC-GlC synapses suggesting serotonergic excitation of the GlC enhancing GABA/glycine release, which in turn facilitates the PC firing resulting from disinhibition at BC/SC-PC and GoC-granule cell (GrC) synapses. NA can excite the GlC, and enhance directly GABA release from a BC/SC to a PC, resulting in entirely the reduction of PC firing rate. **(A)** is modified from Dieudonné (2001); Simat et al. (2007) and Hirono et al. (2012). **(B)** is from Hirono et al. (2017).

Lugaro/globular cells make a transverse microcircuit within the cerebellar cortex that contains not only PC-Lugaro/globular cell synapses but also other synapses of Lugaro/globular cells to basket/stellate cells, which in turn inhibit PCs, and to Golgi cell-granule cell contacts (Dieudonné, 2001; Simat et al., 2007; Hirono et al., 2012; Miyazaki et al., 2020; Figure 2A). The basal firing levels of PCs regulates the membrane potential of Lugaro/globular cells via a PC-Lugaro/globular cell feedback loop. The firing of Lugaro/globular cells, evoked by glutamatergic or monoaminergic synaptic inputs, thus, causes rectifying the PC firing (Strahlendorf et al., 1984; Hirono et al., 2012; Prestori et al., 2019). A recent morphological study also proposed that Lugaro cells can disinhibit cerebellar cortical activities in a compartment-dependent manner (Miyazaki et al., 2020). Therefore, the Lugaro/globular cell-incorporated microcircuit can synchronize the firing of PCs among different microzones and presumably play a significant role in cerebellar motor control leading to the optimization of multiple muscle activity in motor tasks.

### Electrophysiological Characteristics of Lugaro/Globular Cells

Identification of Lugaro/globular cells under Nomarski optical microscopy is difficult because of their low number and the size of their cell body being similar to that of Golgi cells. Whole-cell patch-clamp recordings, however, were applied to them in acute slices of the rodent cerebellum. These electrophysiological studies revealed that Lugaro/globular cells are normally quiescent but get excited and exhibit robust firing following administration of 5-HT (Dieudonné and Dumoulin, 2000; Dumoulin et al., 2001; Dean et al., 2003; Hirono et al., 2012). This evidence suggests that these cells are the main targets of 5-HT released from serotonergic beaded afferent fibers in the cerebellar cortex. Lugaro/globular cells exhibit inhibitory synaptic currents at higher frequencies compared to Golgi cells (Dieudonné, 2001; Hirono et al., 2012, 2017), since their somata and proximal dendrites are enwrapped by calbindin-positive boutons, meaning they are synaptic inputs from PCs (Palay and Chan-Palay, 1974; Lainé and Axelrad, 2002; Crook et al., 2007; Simat et al., 2007). Electrophysiological and optogenetic approaches have demonstrated that PCs form direct and GABAergic monosynapses onto Lugaro/globular cells, meaning that outputs from several PCs inhibit them (Hirono et al., 2012; Witter et al., 2016). By contrast, fast glutamatergic excitatory synaptic inputs onto Lugaro/globular cells show depression by paired-pulse stimulation only at short interpulse intervals (< 100 ms), meaning that they receive mossy fiber inputs (Dieudonné, 2001; Hirono et al., 2012). Other synaptic contacts to Lugaro/globular cells, such as climbing fibers, granule cell fibers, Golgi cell axons, and Lugaro cell axons have been demonstrated morphologically (Miyazaki et al., 2020) but not yet physiologically.

### Neuromodulation of GABAergic Transmission Between PCs and Lugaro/Globular Cells

5-HT released from serotonergic dense plexuses in the granule cell layer and the PC layer could control the excitability of Lugaro/globular cells by acting on their pre- and post-synaptic sites. The 5-HT-mediated membrane depolarization of Lugaro/globular cells is likely attributed to the activation of 5-HT_2_ or 5-HT_6_ receptors postsynaptically (Dieudonné, 2001). Our recent evidence indicates that globular cells are depolarized through the activation of 5-HT_2__*A*_ receptors, which facilitates phosphatidylinositol turnover, but not 5-HT_6_ receptors (Hirono et al., 2017). On the other hand, 5-HT and agonists for 5-HT_1__*B*_ receptors exert a reduction in GABA release from PCs to globular cells, indicating that 5-HT_1__*B*_ receptors are expressed on presynaptic terminals of PC axon collaterals (Hirono et al., 2017; Table 1). Because 5-HT_1__*B*_ receptors are normally coupled to the G protein (G_*i/o*_), which negatively regulates adenylyl cyclase, the 5-HT-mediated inhibitory effects could be attributed to a decrease in phosphorylation levels of proteins associated with synaptic transmitter release. This indicates that 5-HT regulates the membrane excitability of PCs and Lugaro/globular cells via the differential modulatory effects on both GABAergic synaptic circuits and the membrane potential.

**TABLE 1:** Monoaminergic modulation of GABAergic transmission onto LC/GIC or large DCN neuron.

		PC-LC/GIC	PC-DCN neuron
5-HT	Pre	↓ 5-HT_1B_ (Hirono et al., 2017)	↓ 5-HT_1B_ (Saitow et al., 2009)
	Post	↑ 5-HT_2A_ (Dieudonné, 2001; Hirono et al., 2017)	↑ 5-HT_2A_, 5-HT_5_ (Saitow et al., 2009; Zhang et al., 2014)
NA	Pre	↓α_2_ (Hirono et al., 2017)	NR
	Post	↑ only GIC α_1_ and/or β_2_ (Hirono et al., 2017)	↓α_2,_ ↑β_2_ (Di Mauro et al., 2013)

*PC, Purkinje cell; LC, Lugaro cell; GIC, globular cell; DCN, deep cerebellar nucleus; NR, not reported.*

Noradrenergic beaded fibers are abundant just below the PC layer (Nelson et al., 1997). Thus, NA is likely released abundantly to cell bodies of Lugaro/globular cells to be more effective. Interestingly, globular, but not Lugaro cells, are excited to elicit firing during administration of NA (Hirono et al., 2012), suggesting that globular cells could express α_1_- and/or β_2_-adrenoceptors similar to molecular layer interneurons (Saitow et al., 2000; Hirono and Obata, 2006). NA also inhibits synaptic release of GABA from the PC axon collateral terminals onto globular cells via α_2_-adrenoceptor activation (Hirono et al., 2017; Table 1). Because the mouse cerebellar cortex expresses α_2__*A*_ and α_2__*B*_, but not α_2__*C*_-adrenoceptors (Talley et al., 1996; Hirono et al., 2008), α_2__*A*_- and/or α_2__*B*_-adrenoceptors located on presynaptic terminals of the PC axon collateral could mediate the noradrenergic attenuation of GABAergic transmission. Previous papers have reported that NA enhances GABAergic inhibition at basket/stellate cell-PC synapses through the activation of presynaptic α_1_- and β-adrenoceptors (Llano and Gerschenfeld, 1993; Cheun and Yeh, 1996; Kondo and Marty, 1998; Mitoma and Konishi, 1999; Saitow et al., 2000; Hirono and Obata, 2006). Thus, soon after PC firing facilitation mediated by the NA-evoked firing of globular cells, PC firing is thought to be reduced by NA-induced enhancement of GABAergic inhibition onto PCs (Figure 2B). Furthermore, in contrast to serotonergic afferent fibers in the cerebellum, which run along the mediolateral direction and influence many microzones (Hökfelt and Fuxe, 1969; Bishop and Ho, 1985; Longley et al., 2020), noradrenergic afferent fibers travel mainly along the rostrocaudal axis in a few microzones (Longley et al., 2020), suggesting that NA-induced firing of globular cells could synchronize PC firing in a few microzones transiently just after NA-evoked globular cell firing. In addition to the control of PC firing mediated by the NA-driven globular cell-incorporated microcircuit, NA modulates excitatory inputs onto PCs as follows: NA enhances parallel fibers and increases spontaneous spike firing of PCs under *in vivo* conditions (Guo A. et al., 2016). The activation of β_2_-adrenoceptors potentiates parallel fiber-PC synaptic transmission, while the activation of α_1_- and α_2_-adrenoceptors depresses the transmission (Lippiello et al., 2015). NA also reduces the glutamate release at climbing fiber-PC synapses via presynaptic α_2_-adrenoceptor activation (Carey and Regehr, 2009). Therefore, NA exerts more complicated effects in the cerebellar cortex through activating various types of adrenoceptors with the affinity for NA: α_2_- (high affinity), α_1_- (intermediate affinity) and β-adrenoceptors (low affinity) (Atzori et al., 2016). As the locus coeruleus project their afferent fibers to the cerebellar cortex (Nelson et al., 1997), firing modes of the locus coeruleus neurons can change the concentration of NA in the cerebellar cortex. In response to the different behavioral states such as sleep, quiet wake, active wake, and fight-or-flight, projection neurons in the locus coeruleus fire in distinct modes (Aston-Jones and Cohen, 2005; Atzori et al., 2016). Thus, at each behavioral state, the locus coeruleus-NA system play a role in fine tuning of PC firing to contribute to adequate cerebellar functions.

## Large Glutamatergic Neurons in the DCN

### GABAergic Transmission Between PCs and Large Glutamatergic DCN Neurons

The DCN, lateral, interpositus and medial, consist of six types of neurons: glutamatergic, GABAergic and two glycinergic projection neurons, and GABAergic or GABAergic/glycinergic and glutamatergic local interneurons (Chan-Palay, 1977; Uusisaari et al., 2007; Uusisaari and Knöpfel, 2011; Ankri et al., 2015). Large glutamatergic DCN neurons provide the sole output of the cerebellum to various brain areas, including the brainstem and thalamus (Kelly and Strick, 2003; Proville et al., 2014). Recent studies have demonstrated that large glutamatergic DCN neurons make monosynaptic transmission to the ventral tegmental area (VTA) (Carta et al., 2019), and that climbing fibers contribute to reward prediction but not error prediction for cerebellar motor learning (Holloway and Lerner, 2019). The VTA is known to project its dopaminergic axons to the frontal cortex which controls reward, motivation, and cognition, suggesting that neuronal activity of the DCN neurons can play a crucial role in higher cognitive functions, as well as in motor learning.

Direct GABAergic inhibition from PCs generates post-hyperpolarization rebound firing of large DCN neurons (Llinás and Mühlethaler, 1988; Aizenman and Linden, 1999; McKay et al., 2005; Baumel et al., 2009; Hoebeek et al., 2010; Bengtsson et al., 2011). Based on the reports that the firing rates of large DCN neurons are regulated in a linear manner by the inhibitory input rate (Gauck and Jaeger, 2000; Shin and De Schutter, 2006; Shin et al., 2007), the synchronicity and the extent of inhibitory PC input from the cerebellar cortex can play a role not only in the rate but also in the temporal precision patterns of firing of DCN neurons. Rebound firing responses depend on the duration and strength of membrane hyperpolarization and are significant for creating cerebellar timing signals (Aizenman and Linden, 1999; Koekkoek et al., 2003). Furthermore, it is involved in the formation of excitatory synaptic long-term potentiation (LTP) of transmission between mossy fibers onto large DCN neurons (Racine et al., 1986; Pugh and Raman, 2006, 2008; Person and Raman, 2010). The LTP has been thought to contribute to facilitating firing of large neurons in the DCN (Wu and Raman, 2017; Yarden-Rabinowitz and Yarom, 2017), and to be one of critical synaptic mechanisms underlying cerebellar motor learning, including ocular reflex adaptation (Miles and Lisberger, 1981; Kassardjian et al., 2005; Shutoh et al., 2006; Okamoto et al., 2011) and delay eyeblink conditioning (Krupa et al., 1993; Medina and Mauk, 1999; Attwell et al., 2002; Christian and Thompson, 2003; Kistler and De Zeeuw, 2003; Wetmore et al., 2008).

### Monoaminergic Modulation of GABAergic Transmission Between PCs and Large Glutamatergic DCN Neurons

Compared to the large number of reports that neural activity in the cerebellar cortex is modulated by 5-HT, there are fewer reports studying serotonergic modulation of neuronal activity of the DCN (Gardette et al., 1987; Kitzman and Bishop, 1997; Di Mauro et al., 2003; Saitow et al., 2009; Murano et al., 2011). Exogenous administration of 5-HT facilitated spontaneous firing in most neurons in the DCN. A study on the interpositus nuclei demonstrated that 5-HT elicits a slow depolarization in their neurons (Saitow et al., 2009), whose underlying mechanism is that the activation of 5-HT_5_ receptors, which are normally coupled to the G_*s*_ proteins, increases intracellular cAMP levels and augments HCN channel activation, leading to the induction of inward currents in large DCN neurons. In the cerebellar fastigial nucleus of adult rats, 5-HT_2__*A*_ receptors are expressed and their activation elicits excitatory effect on neurons (Zhang et al., 2014; Table 1). These discrepancies are likely caused by differences in experimental aspects such as the contribution of synaptic transmission regulating neuronal excitability, the experimental sample (brain slices vs. *in vivo*), and the animal age (juvenile vs. adults). By contrast, 5-HT applied microiontophoretically suppressed firing of neurons of the medial nucleus and caused complicated effects (inhibitory, excitatory, and biphasic) on neurons not only of the interpositus but also of lateral nuclei (Di Mauro et al., 2003). As described below, 5-HT evokes slow excitatory inward currents postsynaptically, which facilitate discharge of DCN neurons, and subsequently attenuate the impact of rebound firing after repetitive inhibition by suppressing the GABA-mediated hyperpolarization. The impact of rebound depolarization is interrupted by 5-HT at relatively high concentrations because the 5-HT-mediated depolarization facilitates significantly the background spikes of DCN neurons. Thus, 5-HT may prevent the induction of LTP at synapses between mossy fibers and large DCN neurons. By contrast, 5-HT also modulates GABA release from presynaptic terminals on large DCN neurons (Saitow et al., 2009). GABAergic transmission is inhibited by the activation of presynaptic 5-HT_1__*B*_ receptors (Table 1).

It is possible that NA released from noradrenergic beaded fibers modulates activity of large DCN neurons. The β-adrenergic agonist isoproterenol can enhance the responses of DCN neurons to GABA applied microiontophoretically (Gould et al., 1997), meaning that β-adrenoceptors are postsynaptically expressed on large DCN neurons and modulate GABA inhibition. Di Mauro et al. (2013) tested the NA-mediated effects on inhibitory GABA responses in neurons of the various DCN (medial, posterior and anterior interpositus, and lateral), and showed that NA levels alter the responsiveness of DCN neurons to GABA. They indicated that NA modifies GABA responses through the activation of likely postsynaptic α_2_- and/or β-adrenoceptors (Table 1). In the inferior vestibular nucleus, NA directly regulates the excitability of neurons by the activation of α_1_-, α_2_- and β_2_-adrenoceptors (Peng et al., 2016). In any case, NA-mediated alteration of neuronal activity in each of these nuclei would have functional significances. Changes in NA concentration, as commonly observed in behavioral state, stress or aging, could influence neural activity of DCN neurons. Whereas spontaneous firing of PCs in each behavioral state is regulated by NA-mediated complex mechanisms as described previously, the NA-mediated effect on GABA release from presynaptic terminals of PC primary axons in the DCN has yet to be tested.

## Physiological Roles of Perineuronal Nets in the DCN

### General Information on Perineuronal Nets

A PNN is the fourth most important element for the tetrapartite synapse in addition to presynapses, postsynapses, and glial cells, and serves as a regulator of synaptic functions and plasticity (Dityatev and Rusakov, 2011; Chelini et al., 2018). The major components of PNNs are CSPGs, which have a core protein with long chondroitin sulfate chains, tenascin-R, link proteins, and hyaluronic acid, and are synthesized by both neurons and glial cells (Oohashi et al., 2015). To date, it has been reported that during brain development, PNNs contribute to the normal maturation of neuronal circuits, including fast-spiking parvalbumin-positive neurons (Cabungcal et al., 2013; Reichelt et al., 2019). PNNs morphologically restrict the production of new synapses and the pruning of old synapses, and contribute to the regulation of neuronal plasticity in various brain areas, such as the visual cortex and the amygdala (Pizzorusso et al., 2002; Gogolla et al., 2009; Carulli et al., 2010; Shen, 2018). To remove PNNs, enzymatic or genetic techniques have been widely adopted, and studies using these methods have demonstrated that PNN deletion enhances the formation of memories by facilitating plasticity and encoding new information that occurs by attenuating forgetting or learning information easily (Sorg et al., 2016; Fawcett et al., 2019; Reichelt et al., 2019). Moreover, PNNs have been focused on as the cause of the pathophysiology of brain disorders. Aberrant PNNs have been reported to be associated with neurodegenerative and neuropsychiatric disorders through abnormal neuroplasticity (Sorg et al., 2016; Suttkus et al., 2016; Wen et al., 2018; Fawcett et al., 2019; Reichelt et al., 2019). By contrast, it has not been elucidated whether PNNs can regulate cerebellar neuronal circuits dynamically and functionally, nor how PNNs contribute to the regulation of motor learning.

### Expression of Perineuronal Nets in the Cerebellum

**FIGURE 3:**
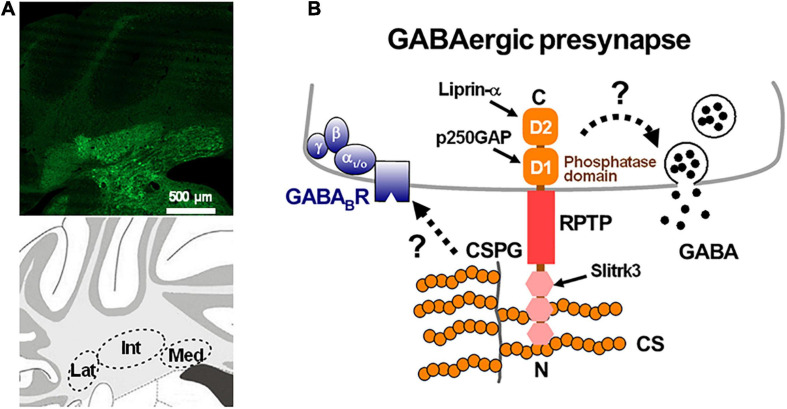
Perineuronal net-expressing in the deep cerebellar nuclei and schematic diagram of a GABAergic presynaptic terminal. **(A)** Perineuronal nets, which are labeled by *Wisteria floribunda agglutinin* (green), are expressed in the deep cerebellar nuclei: interpositus (Int), lateral (Lat) and medial (Med) nucleus of a mouse coronal section (upper panel). Schematic view of the three nuclei in the mouse cerebellar coronal section (lower panel). **(B)** A chondroitin sulfate proteoglycan (CSPG) binds to receptor-type protein tyrosine phosphatase (RPTP) via the chondroitin sulfate binding N-terminal of the RPTP. The intracellular domains of the RPTP may alter the release of GABA by directly controlling the release machinery or the intracellular Ca^2+^ concentration through changes of the phosphatase activity, or by interacting with Slitrk3, p250GAP, and liprin-α (Südhof, 2012). The CSPG may also regulate the expression of presynaptic GABA_*B*_ receptors (Dzyubenko et al., 2020). (A) is our unpublished data.

Perineuronal nets are detected in the brain using labeled *Wisteria floribunda agglutinin* (WFA), which is a lectin that recognizes the *N*-acetylgalactosamine segments of sugar chains in PNNs. In many brain regions, PNNs preferentially enwrap inhibitory parvalbumin-positive neurons, which are highly active and involved in critical periods of brain development, and play a crucial role in the regulation of neuronal functions and synaptic plasticity (Cabungcal et al., 2013; Fawcett et al., 2019; Reichelt et al., 2019). In the cerebellar cortex, Lugaro/globular cells, which receive strong GABAergic inhibition from PCs via their axon collaterals, are recognized by a monoclonal antibody against aggrecan, Cat-301 (Sahin and Hockfield, 1990; Zaremba et al., 1990; Crook et al., 2007), which means that they are surrounded by PNNs. On the other hand, both the cell bodies and the proximal dendrites of large and excitatory DCN neurons, which are primarily glutamatergic (Telgkamp and Raman, 2002; Uusisaari et al., 2007), are surrounded by PNNs (Figures 1C, 3A) in which aggrecan is the primary CSPG (Zaremba et al., 1990; Carulli et al., 2004, 2006, 2007; Zimmermann and Dours-Zimmermann, 2008; Foscarin et al., 2011; Bekku et al., 2012). Later, we will focus on the physiologically significant roles of PNNs surrounding large glutamatergic DCN neurons in dynamic regulation of GABAergic transmission as well as in cerebellar motor learning.

### Functional Roles of PNNs in GABAergic Transmission Between PC and Large Glutamatergic DCN Neuron

To examine the role of PNNs, techniques for the pharmacological or genetic removal of PNNs have been used. Chondroitinase ABC (ChABC) is a commonly used enzyme that degrades chondroitin sulfate glycosaminoglycans (GAGs). The DCN acutely treated with ChABC indicated a significant reduction in the intensity of WFA labeling by 30% around large DCN neurons and showed potentiates presynaptic release of GABA (Hirono et al., 2018; Table 2). Under the lower density of PNNs, a decrease in the amplitude of inhibitory postsynaptic currents (IPSCs) evoked by a high frequent repetitive stimulation is steeper, which is similar to that observed in the DCN of juvenile mice (Turecek et al., 2016; Saitow et al., 2018), reflecting a high tendency for neuronal plasticity and cerebellar motor learning compared to adults. On the other hand, stable overexpression of ChABC for a long time in the DCN through a lentiviral vector (LV) removed 90% PNNs from large DCN neurons (Carulli et al., 2020). The LV-ChABC mice showed that chronic ChABC treatment evoked the configuration of new GABAergic terminals and a decrease in expression of vesicular glutamate transporter (VGLUT) 1 in the DCN, leading to the attenuation of the spontaneous firing of large DCN neurons (Table 2).

**TABLE 2:** Comparisons of phenotypes in the mouse DCN treated with ChABC.

Characteristic	ChABC treatment (pharmacologically) (Hirono et al., 2018)	ChABC expression (genetically) (Carulli et al., 2020)
Effect	Acute	Chronic
WFA intensity	30% Reduction	90% Reduction
Morphology of GABAergic terminals	No change	Smaller and Increase
Morphology of glutamatergic terminals	NR	VGLUT1 Decrease
Electrophysiological characterization of DCN neurons	No change of RMP	Lower spike activity
GABAergic transmission	Potentiation	NR
Behavioral characterization (EBC)	Facilitation	Facilitation

*EBC, eyeblink conditioning; NR, not reported; RMP, resting membrane potential.*

Manipulation of specific genes related to PNNs can resolve PNN components. In mice lack of each PNN component such as HAPLN1, HAPLN4, and tenascin-C, the number of PC terminals on large neurons in the DCN was reported to be affected (Bekku et al., 2003, 2012; Foscarin et al., 2011; Stamenkovic et al., 2017; Edamatsu et al., 2018). By contrast, mouse cerebellar slices acutely treated with ChABC did not show anatomical changes of synapses on large DCN neurons; thus, acute enzymatic removal of PNNs did not change the size or number of PC axon terminals on large DCN neurons (Hirono et al., 2018). Thus, ChABC-mediated acute removal of PNNs by enhances the release probability of GABA rather than the new formation of presynaptic terminals of PC axons. Similarly, in the hippocampus, after treatment with ChABC for 24 h, CA1 pyramidal cells also showed an increase in the frequency of sIPSCs without changing the amplitude, and a theta burst stimulation-induced long-term plasticity in GABAergic transmission in the opposite way (Shi et al., 2019). Thus, it is concluded that PNNs can control presynaptic functions of PC axons in the DCN, and modulate synaptic plasticity and membrane excitation of large neurons in the DCN.

### Potential Mechanisms Underlying PNN Regulation of Presynaptic GABA Release

There are likely four potential mechanisms underlying the augmentation of GABA release from terminals of PC axons with acute ChABC-mediated digestion of PNNs: first, releasing the limitation of extracellular Ca^2+^ availability, second, altering the conductance of presynaptic voltage-dependent Ca^2+^ channels, third, facilitating the release machinery of GABA, and fourth, augmenting the presynaptic basal intracellular Ca^2+^ concentrations in PC axon terminals. The most possible mechanism could be that negatively charged CSPGs buffer Ca^2+^ through interacting electrically (Crank, 1975) and confine the efficiency of extracellular Ca^2+^ around PC axon terminals (Hrabětová et al., 2009; Nicholson and Hrabětová, 2017). The extracellular matrix slows down Ca^2+^ diffusion around synapses, with this effect being attenuated by ChABC-mediated PNN deletion. However, the ChABC-mediated GABA release facilitation was not affected either by alterations in extracellular Ca^2+^ concentrations or by inhibition of voltage-dependent Ca^2+^ channels (Hirono et al., 2018). Thus, the latter two explanations described above could be plausible. PNN degradation could facilitate the release machinery by enhancement of its Ca^2+^ sensitivity and/or the intrinsic activity, or actuation of intracellular Ca^2+^ release in terminals of PC axons. CSPG removal could change the activity of type IIa receptor-type protein tyrosine phosphatases (RPTPs), leukocyte common antigen-related (LAR) proteins, PTPσ, and PTPδ, because they bind to CSPG-chondroitin sulfates (Shen et al., 2009; Fisher et al., 2011). CSPGs are known to control PTPσ and LAR receptors since ablation of the carbohydrate chains of CSPGs by ChABC causes regeneration of axons by the inactivation of PTPσ through its clustering (Coles et al., 2015; Lang et al., 2015). Cerebellar PCs are reported to express PTPσ and NgR3, which bind to CSPG-chondroitin sulfates (Funahashi et al., 2008; Brown et al., 2020). Therefore, ChABC-mediated chondroitin sulfate digestion, which can inactivate these receptors, likely change the balance between tyrosine phosphorylation and dephosphorylation, resulting in facilitating the release machinery for GABA release (Figure 3B). Recently, an alternative mechanism has been proposed that PNN depletion by ChABC administration reduces presynaptic GABA_*B*_ receptors (Dzyubenko et al., 2020). Further investigations are needed to identify the exact molecular mechanism of the PNN-mediated modulation of presynaptic GABA release.

Large DCN neurons display post-inhibitory rebound firing after the relief of hyperpolarization induced by GABAergic transmission from PCs. Acute *in vitro* PNN depletion by ChABC treatment remarkably enhanced inhibitory GABAergic transmission between PCs and large DCN neurons, which induced an augmentation of hyperpolarization, thus, facilitating the rebound firing in the neurons, meaning improvements in cerebellar motor learning, as described in the next section (Hirono et al., 2018). *In vivo* electrophysiological recordings from neurons in the interpositus nuclei of LV-ChABC mice indicated that chronic PNN deletion markedly suppressed the baseline activity of the neurons (Carulli et al., 2020). They suggested that this firing reduction of DCN neurons could reduce the level of rebound firing in DCN neurons at the time of expression of conditioned responses (CRs) seen during delay eyeblink conditioning (ten Brinke et al., 2017). On the other hand, the LV-ChABC mice also demonstrated an increase in the density of GABAergic terminals around DCN neurons. Thus, the enhanced inhibition could facilitate the rebound firing as observed in the cerebellar slices acutely treated with ChABC.

### PNNs in the Interpositus Nuclei Control Motor Learning of Delay Eyeblink Conditioning

Chondroitinase ABC was directly injected into various brain areas, and *in vivo* experiments with PNN deletion demonstrated the significance of PNNs in brain functions such as learning and memory (Pizzorusso et al., 2002; Corvetti and Rossi, 2005; Gogolla et al., 2009; de Vivo et al., 2013; Romberg et al., 2013; Banerjee et al., 2017). Inactivation of the DCN via lesions or pharmacological treatments has been reported to restrict the increases in CRs of trained mice (Yeo et al., 1985; Attwell et al., 2002; Ohyama et al., 2006; Boele et al., 2010; Sakamoto and Endo, 2010). Additionally, the activation of the interpositus nuclei is required for obtaining CRs during delay eyeblink conditioning (Heiney et al., 2014; ten Brinke et al., 2017). Therefore, the roles of PNNs expressed in the interpositus nuclei in delay eyeblink conditioning were tested pharmacologically and genetically by PNN digestion. Both the ChABC-injected and LV-ChABC mice exhibited significant enhancements in their CR rates, suggesting that PNN depletion in the interpositus nuclei facilitates CR acquisition (Hirono et al., 2018; Carulli et al., 2020). This facilitation of motor learning is similar to observations in other brain regions, where PNN loss leads to synaptic reorganization structurally and facilitates memory formation in adulthood (Corvetti and Rossi, 2005; Gogolla et al., 2009; Romberg et al., 2013; de Vivo et al., 2013), however, a novel role of PNNs in controlling GABA release from presynaptic terminals in the DCN is proposed, and a novel mechanistic insight for the functional synaptic plasticity regulated by PNNs has been demonstrated to influence behavioral flexibility in adulthood (Hirono et al., 2018). The developmental increase in PNN expression suppresses GABA release from PC axons to large DCN neurons and disturbs new formation of memories. This developmental change may also conserve cerebellar memories encoded before adulthood. Intriguingly, a reduction of PNNs in the DCN is observed during the acquisition and consolidation of eyeblink conditioning of adult mice (Carulli et al., 2020). These findings demonstrate that modulation of PNNs is significant for the dynamic regulation of GABAergic transmission in the DCN and for fine control of cerebellar motor learning.

### Regulation of PNN Density by Voluntary Exercise

**FIGURE 4:**
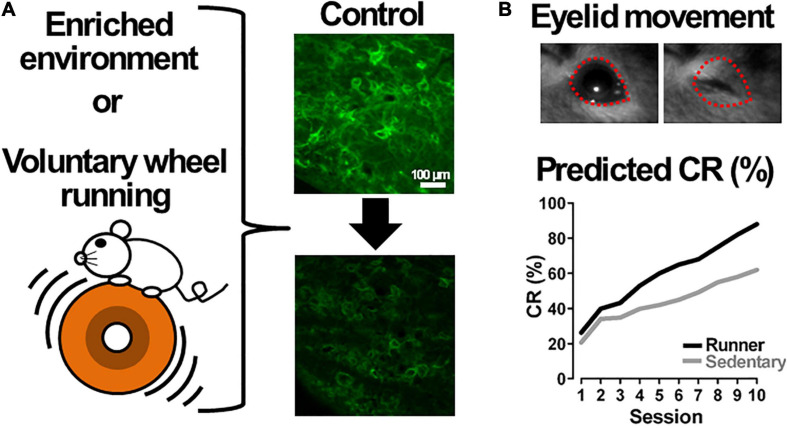
Voluntary exercise in an enriched environment or on a running wheel could facilitate cerebellar motor learning. **(A)** A mouse exercises in an enriched environment (EE) or on a running wheel. EE or voluntary wheel running likely reduces the density of perineuronal nets (right panels): the upper image shows large neurons surrounded by perineuronal nets labeled by *Wisteria floribunda agglutinin* (green) in the mouse deep cerebellar nucleus, and the lower shows a predicted image from a mouse kept in an EE or on a running wheel. The upper image is our unpublished data. **(B)** Voluntary exercise could improve conditioned responses (CRs) of cerebellar motor learning in delay eyeblink conditioning. The upper image is an example of eyelid movement during mouse delay eyeblink conditioning (Hirono et al., 2018). The lower graph shows predicted learning curves obtained from a mouse kept with (Runner) or without (Sedentary) a running wheel, respectively.

As described above, degradation of PNNs in the DCN enhances cerebellar motor learning. Additionally, a reduction in PNNs surrounding large DCN neurons occurs during the acquisition and consolidation of eyeblink conditioning (Carulli et al., 2020). These findings lead us to suggest that environmental stimuli could be useful in facilitating cerebellar functions including motor learning and cerebellum-mediated cognitive functions, because it was proposed that animals exposed to an enriched environment (EE) show decrease in PNNs in the CNS (Sale et al., 2007; Madinier et al., 2014), leading to facilitate neural plasticity. The EE has been suggested to enhance learning, facilitate recovery from brain lesions and brain disease, and postpone age-dependent cognitive decline (Nithianantharajah and Hannan, 2006; Pang and Hannan, 2013; Hirase and Shinohara, 2014). Indeed, mice exposed to an EE have also demonstrated a reduction in PNNs in the DCN (Foscarin et al., 2011; Stamenkovic et al., 2017). Foscarin et al. (2011) reported that the EE decreased the expression of mRNAs coding for key PNN molecules, and enhanced the activity of matrix degrading enzymes matrix metalloproteinases 2 (MMP-2) and MMP-9, via a dynamic interaction between PC axons and DCN neurons. It is possible that these mice could exhibit the CR at a significantly higher rate in delay eyeblink conditioning, suggesting an improvement in their motor performance (Figure 4). This expectation could be supported by the evidence that acquisition and expression of learning of delay eyeblink conditioning in mice are enhanced in a locomotor activity-dependent manner on a running wheel (Albergaria et al., 2018). They suggest that the enhanced eyeblink conditioning could be attributed to facilitation of the mossy fiber-granule cell connection by locomotor activity, however, there is another likely reason that the locomotor activity could reduce PNN expression in the DCN and improve cerebellar motor learning (Figure 4).

Increased motor activity such as voluntary wheel running can reduce the PNN expression. In rat experiments, free access to a running wheel caused PNN deletion in the hippocampus, but not in the DCN (Smith et al., 2015). Thus, additional studies in which mice are exposed to a running wheel are required, and a correlation between the running distances and the PNN expression levels in the DCN should be demonstrated. Considering that the voluntary wheel running evokes structural plasticity of the NA system: shortened inter-varicosity intervals in the cerebellum (Nedelescu et al., 2017), it is still possible that voluntary exercises in an EE and on a running wheel can improve not only cerebellar motor learning but also cognitive functions through several neuronal mechanisms, presumably including alternations of PNN density in the DCN.

## Conclusion and Future Directions

A variety of neuronal modulators are involved in controlling cerebellar neural circuits. The characterization of the modulation of synaptic transmission and intrinsic neuronal excitation is significant for a precise comprehension of cerebellar motor learning and cognitive processing depending on the cerebellum. PCs project not only their primary axons to the DCN but also collaterals within the cerebellar cortex, especially around the PC layer to form negative feedback connections. Both the GABAergic transmission from PCs is important because the primary axons transmit signals, which are formed by PCs after integrating excitatory and inhibitory input signals, and the axon collaterals form Lugaro/globular cell-incorporated microcircuits. Thus, we focused on the characteristics of PC-mediated GABAergic transmission and the modulatory effects of monoamines and PNNs on synaptic transmission.

5-HT excites Lugaro/globular cells and large DCN neurons through pre- and post-synaptic mechanisms. The activation of presynaptic 5-HT_1__*B*_ suppresses the release of GABA from PC axon terminals, and the activation of 5-HT_2__*A*_ receptors (and 5-HT_5_ receptors for large DCN neurons) depolarizes the membrane potential of postsynaptic neurons. Thus, 5-HT released in the cerebellar cortex activates the Lugaro/globular cell-incorporated microcircuit, facilitating and synchronizing the activity of PC clusters, followed by a return to the basal activity of Lugaro/globular cells. 5-HT-evoked excitatory effects on large glutamatergic DCN neurons could be independent of the 5-HT-mediated activation of the microcircuit in the cerebellar cortex, because serotonergic fibers from various brainstem nuclei innervate separately into the cerebellar cortex and DCN (Kitzman and Bishop, 1994). In the cerebellar cortex, NA postsynaptically excites globular cells but not Lugaro cells presumably via the activation of α_1_- and/or β_2_-adrenoceptors. This postsynaptic excitation could be reinforced by a NA-mediated presynaptic effect, which is a NA-mediated inhibition of GABA release from PC axon collaterals through α_2__*A*_ and/or α_2__*B*_–adrenoceptor activation. Thus, NA-mediated activation of a globular cell-incorporated microcircuit could also synchronize PC firing, followed by inhibition caused by NA-evoked excitation of basket/stellate cells. However, little is known regarding the direct effects of NA on the membrane potential of the large DCN neurons and GABA release from the terminals of PC primary axons. It has been reported that postsynaptic activation of α_2_- and β-adrenoceptors enhanced and decreased GABA responses, respectively. Further studies will be needed to determine NA-mediated direct effects on pre- and post-synaptic sites between PC-large DCN neuron synapses. Awake animals show sustained discharge of noradrenergic neurons in the locus coeruleus and of serotonergic neurons in the raphe nuclei. Animals in more active conditions such as in an aspect of active waking, alertness, or stress exhibit facilitation of firing of monoaminergic neurons. Previous studies demonstrated several relationships between the firing of these monoaminergic neurons and motor activity (Jacobs et al., 1991; Veasey et al., 1995, 1997; Mendlin et al., 1996). It will be of importance to demonstrate that in active animals, the excitatory effects of 5-HT and NA on Lugaro/globular cells can contribute to motor coordination of the cerebellum additively or synergistically.

In the CNS, PNNs, which predominantly wrap inhibitory parvalbumin-positive neurons, regulate synaptic plasticity and neuronal functions and are involved in controlling various brain functions such as learning and memory. PNN digestion facilitates GABAergic transmission at PC-large DCN neuron synapses. Acute pharmacological PNN removal functionally facilitates GABA release from presynaptic PC terminals, whereas chronic PNN removal increases the number of PC terminals on large DCN neurons. Mice that received ChABC pharmacologically or genetically in the interpositus nuclei showed CRs in delay eyeblink conditioning at a higher rate than that of control mice. This evidence suggests that PNN alteration makes memories flexible or consolidated, thereby affecting the flexibility of functions of the mature cerebellum. Whereas Lugaro/globular cells are known to be parvalbumin-negative, large glutamatergic DCN neurons surrounded by PNNs may be parvalbumin-positive neurons because it has been reported that parvalbumin-positive large bipolar neurons are present in the DCN (Bastianelli, 2003) and their synaptic dysfunction generates an action tremor (Zhou et al., 2020). As PNNs regulate spontaneous firing of large glutamatergic DCN neurons, which project their axons to various brain areas such as the brainstem, thalamus, and VTA, alternations in PNN density in the DCN can regulate not only cerebellar motor learning but also cognitive functions that depend on the cerebellum. Thus, future studies on mechanisms underlying the manipulation of specific components in the PNNs formed in the DCN will be important to unravel the physiology of cerebellar motor learning and cognitive processing.

Whereas PNNs in the mature CNS are known to restrict neuronal plasticity, a reduction in PNNs has been observed in multiple brain disorders, including cognitive dysfunction (Paylor et al., 2018), depression (Yu et al., 2020), Alzheimer’s (Bruckner et al., 1999; Baig et al., 2005; Morawski et al., 2010; Crapser et al., 2020), epilepsy (McRae and Porter, 2012; Pollock et al., 2014; Tewari et al., 2018; Chelyshev et al., 2020), schizophrenia (Pantazopoulos et al., 2010; Mauney et al., 2013; Enwright et al., 2016; Steullet et al., 2017), and bipolar disorders (Testa et al., 2019). Thus, to elucidate mechanisms underlying the regulation of PNN density related to neuronal plasticity in the CNS (Zaki and Cai, 2020) could be useful for finding out effective treatments for neurological disorders and psychiatric illnesses.

## Author Contributions

MH, FK, and YY designed the project of this review, carried out the literature search and analysis, and wrote and edited the manuscript. All authors contributed to the article and approved the submitted version.

## Conflict of Interest

The authors declare that the research was conducted in the absence of any commercial or financial relationships that could be construed as a potential conflict of interest.
